# Synergistic photothermal antibacterial efficacy and high biocompatibility of silver-carbon core-shell nanoparticles

**DOI:** 10.1186/s11671-026-04432-w

**Published:** 2026-01-16

**Authors:** Chunning Gu, Li Guo, Ziqian Zhou, Anyuan Shi, Lele Wu, Wei Cheng

**Affiliations:** Nanjing Stomatological Hospital, Affiliated Hospital of Medical School, Institute of Stomatology, Nanjing University, 30 Zhongyang Road, Nanjing, 210008, Jiangsu China

**Keywords:** Silver-based nanoparticles, Core-shell nanoparticles, Antibacterial, Photothermal therapy, Biocompatibility

## Abstract

Safe and effective antimicrobial treatment strategies are urgently required for the prevention and control of infectious diseases. While silver-based nanoparticles (AgNPs) are currently acknowledged as the most potent metal-based antibacterial agents, their potential cytotoxicity poses a significant barrier to further clinical applications. Herein, we synthesized carbonaceous coated silver nanocore (Ag@C) core-shell nanoparticles and investigated their material properties, biocompatibility, and antibacterial efficacy. The produced Ag@C exhibited a uniform core-shell structure with an overall diameter of 256.40 nm, a shell thickness of 92.20 nm, and a silver core diameter of 67.45 nm. Under irradiation with 808 nm near-infrared (NIR) irradiation, Ag@C demonstrated excellent photothermal conversion efficiency. The results from apoptosis detection via flow cytometry, CCK-8 cytotoxicity assays, and live/dead cell staining using Calcein-AM/PI, collectively indicated that Ag@C displayed no significant cytotoxicity. Hemolysis tests further confirmed the good biocompatibility of Ag@C. Quantitative analysis through plate counting assays revealed that Methicillin-resistant Staphylococcus aureus (MRSA) co-cultured with Ag@C were significantly eradicated by NIR irradiation; this finding was corroborated by bacterial live/dead staining observed under confocal laser scanning microscope (CLSM). Our results indicate that Ag@C combined with photothermal therapy (PTT) exhibits substantial antibacterial effects *in vitro* while maintaining high biosafety standards, suggesting promising prospects for clinical application.

**Supplementary Information:**

The online version contains supplementary material available at 10.1186/s11671-026-04432-w.

## Introduction

Bacterial infections remain a significant global concern. Statistics indicate that in 2019, approximately 4.95 million deaths were attributed to infections caused by antibiotic-resistant bacteria, with projections suggesting that by 2050, around 10 million individuals will succumb annually to such infections [1, 2]. The rapid advancements in nanobiology have led to innovative research utilizing nanomaterials for antimicrobial therapy. Nanomaterials possess unique physicochemical properties, including small particle size, large specific surface area, high surface energy, customizable shapes, and the potential for surface modification [3]. Given their ability to bypass existing bacterial resistance mechanisms, nanomaterials are less likely to induce bacterial resistance compared to traditional antibiotics [4]. They can function both as direct antibacterial agents and as carriers for antibacterial drugs. Furthermore, several emerging therapeutic strategies based on nanomaterials—such as chemodynamic therapy, photothermal therapy (PTT), magnetic thermal therapy, photodynamic therapy, gas therapy, sonodynamic therapy, and magnetically mediated mechanical therapy—have been successfully applied in the realm of antibacterial treatment [5]. These approaches have yielded satisfactory results; thus, nanomaterials offer numerous effective solutions for addressing drug-resistant bacterial infections and herald a new era in the management of chronic recurrent infections.

Among these antibacterial strategies, PTT has emerged as a particularly promising modality owing to its high selectivity, low invasiveness, and minimal off-target effects [6]. PTT hinges on photothermal agents that convert light energy into localized hyperthermia (42–45 °C), which selectively ablates bacteria while sparing healthy tissues [7]. Laser wavelength selection is a pivotal determinant of PTT efficacy, as it directly governs tissue penetration depth, photothermal conversion efficiency, and biological safety. In clinical antibacterial settings, the 808 nm near-infrared (NIR) laser represents the optimal candidate. This wavelength lies within the “NIR tissue optical window” (700–1100 nm), a spectral range where biological tissues display minimal absorption and scattering [8]. Unlike shorter wavelengths (e.g., 650 nm visible light), which are strongly absorbed by hemoglobin and penetrate merely 0.5–2 cm, or longer wavelengths (e.g., 980 nm) that are susceptible to water absorption-induced systemic hyperthermia, the 808 nm laser penetrates 3–5 cm into deep tissues while retaining effective power density [9]. This attribute is pivotal for targeting deep-seated infections refractory to conventional therapies. Nevertheless, PTT exerts a transient antibacterial effect. To attain sustained antibacterial activity, photothermal agents must inherently possess intrinsic antibacterial properties.

Silver-based nanoparticles (AgNPs) are currently recognized as the most developed metallic antibacterial agents, having been extensively studied and utilized over the past few decades [10, 11]. Although the precise antibacterial mechanism of AgNPs remains incompletely understood, existing research indicates that silver ions (Ag^+^) released from these nanoparticles play a crucial role in their antimicrobial activity [12]. Specifically, Ag^+^ can bind to bacterial thiolases, resulting in their inactivation, which subsequently disrupts microbial energy metabolism and cell wall synthesis [13]. Furthermore, studies have demonstrated that AgNPs can interact with the DNA bases of pathogens, leading to DNA denaturation and thereby affecting genetic material replication [14, 15]. Nevertheless, AgNPs exhibit cytotoxic effects on mammalian cells, presenting a double-edged sword that limits their further application *in vivo *[16]. Moreover, bare AgNPs rely solely on Ag⁺-mediated mechanisms, which are insufficient against high-level drug-resistant pathogens due to limited action modes. This monomodal antibacterial activity lack responsiveness to external stimuli, including light, ultrasound, pH level, that could enhance targeting and efficacy [17]. Given these challenges, a surface modification strategy was urgently needed to simultaneously mitigate cytotoxicity and enhance antibacterial efficacy for promoting the broader use of AgNPs in clinical practice for bacterial infectious diseases.

**Fig. 1:**
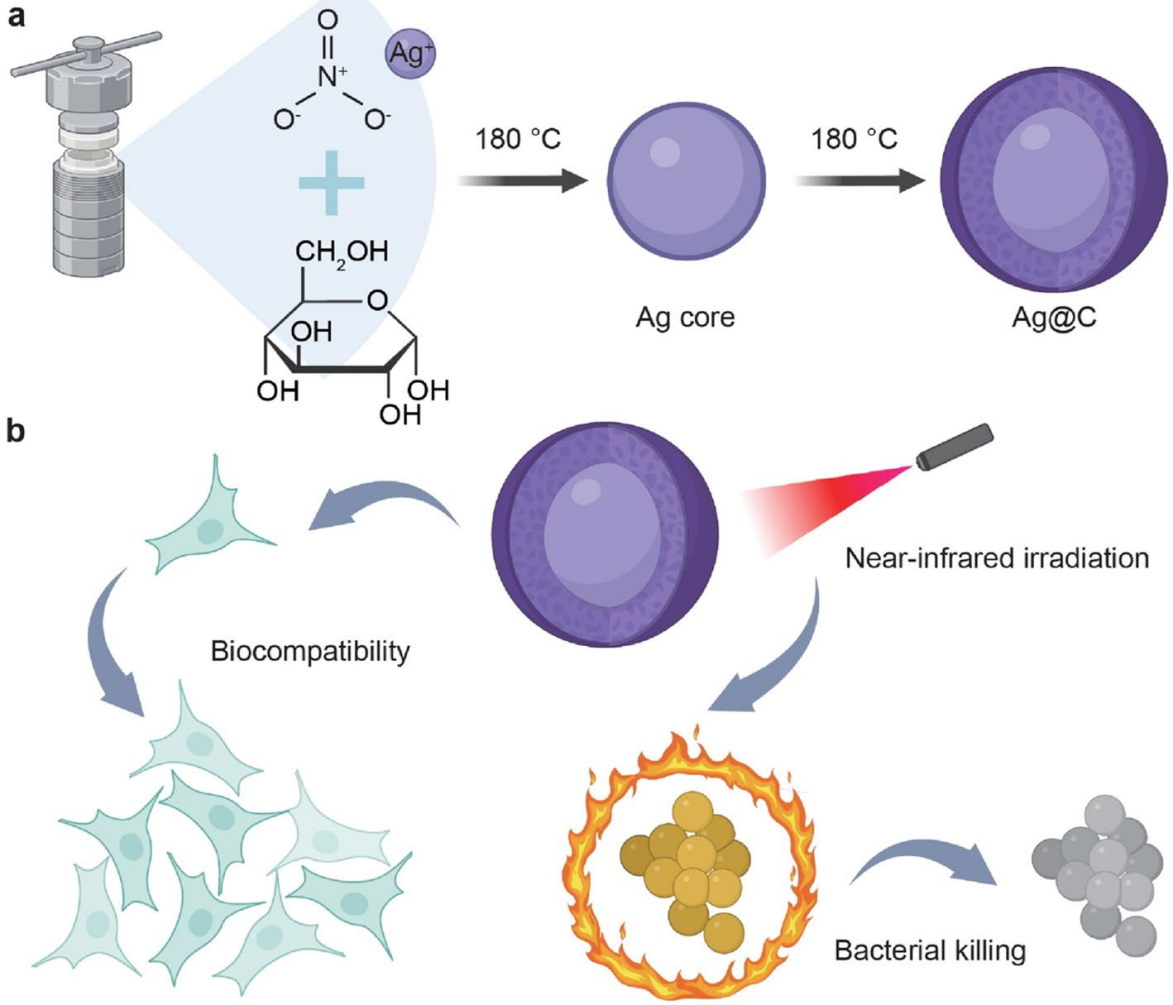
**a** Schematic of the synthesis of the Ag@C core-shell structure nanospheres. **b** The Ag@C core-shell nanospheres demonstrate excellent biocompatibility and are capable of effectively eliminating bacteria through photothermal effects

Carbon-based nanomaterials, including graphene, carbon nanotubes and carbon dots, have emerged as prominent candidates in the antibacterial field owing to their high specific surface area, modifiability and unique antibacterial mechanisms [18]. Already deployed in medical dressings [19], device coatings [20], and dental infections [21], they represent potential alternatives to conventional antibiotics. Despite the prominent antibacterial activity of AgNPs, their clinical translation is hindered by inherent limitations that demand targeted modification—herein, carbon coating emerges as a rational and necessary strategy. The core requirements for introducing a carbon shell stem directly from the unmet needs of bare AgNPs: first, to mitigate the cytotoxicity caused by uncontrolled release of Ag^+^ ions, which accumulate in mammalian tissues and disrupt normal cellular functions; second, to overcome the monomodal antibacterial mechanism of AgNPs (solely dependent on Ag^+^), which is insufficient against high-level drug-resistant pathogens and lacks responsiveness to external stimuli for enhanced targeting.In this study, we prepared carbonaceous coated silver nanocore (Ag@C) core-shell nanoparticles to investigate their fundamental material properties, 808 nm near-infrared (NIR) responsive photothermal characteristics, biocompatibility, and antibacterial efficacy (Fig. 1). This study aims to synthesize and characterize Ag@C, evaluate its biocompatibility, study its synergistic antibacterial effect with NIR-photothermal, and verify its structural advantages for clinical use. The results indicated that the Ag@C core-shell nanoparticles exhibit a uniform morphology, demonstrating good biocompatibility and an excellent synergistic response to NIR irradiation along with efficient antibacterial performance. Consequently, Ag@C core-shell nanoparticles effectively balance the antibacterial properties and biocompatibility of silver nanoparticles (AgNPs), presenting a promising option for clinical antibacterial therapy.

## Materials and methods

### Chemicals and materials

Glucose and silver nitrate (AgNO_3_) were sourced from Sinopharm Chemical Reagent (Shanghai, China). Dulbecco’s modified Eagle’s medium (DMEM), along with penicillin sulfate and streptomycin (P/S), was acquired from HyClone (UT, USA). Fetal bovine serum (FBS) and trypsin-EDTA solution were obtained from Gibco (CA, USA). Cell counting kit-8 (CCK-8), calcein/PI cell viability/cytotoxicity assay kit, and Annexin V-FITC/PI apoptosis kit were procured from Dojindo (Kumamoto, Japan), Beyotime (Shanghai, China), and MultiSciences (Hangzhou, China), respectively.

### Synthesis of Ag@C core-shell nanoparticles

Ag@C core-shell nanoparticles were prepared based on using the method previously reported [22]. Briefly, AgNO_3_ aqueous solution (0.1 M, 0.5 mL) was gradually added dropwise to 35 mL of glucose solution (100 mg/mL) while maintaining vigorous stirring. After a stirring period of 10 min, the reaction mixture was transferred to a Teflon-lined autoclave and maintained at 180 °C for 4 h. Subsequently, the mixture was permitted to cool down to ambient temperature on its own. The resulting product was separated from the reaction medium through centrifugation at 10,000 rpm for a duration of 10 min. Following this, it underwent three washing and redispersion cycles in deionized water and alcohol to ensure thorough purification, before being dried in an oven at 80 °C for 4 h.

### Characterization of nanoparticles

Scanning electron microscopy (SEM) images were recorded using a Hitachi SU8010 microscope (Hitachi; Tokyo, Japan) set to an operating voltage of 5 kV. Additionally, transmission electron microscopy (TEM) and elemental mapping images were acquired with a JEOL JEM 2100 F microscope (JEOL; Tokyo, Japan), functioning at an acceleration voltage of 200 kV. X-ray photoelectron spectroscopy (XPS) analyses were performed utilizing a PHI 5000 VersaProbe (ULVAC-PHI, Chigasaki, Japan). Ultraviolet-visible-near-infrared (UV-Vis-NIR) absorbance spectrum were recorded using a PerkinElmer Lambda 365 spectrometer (PerkinElmer, Billerica, MA, USA). Concurrently, a commercial 808 nm NIR semiconductor laser (Changchun New Industries Optoelectronics Technology Co., Ltd., China) and a NIR thermal imager (FLIR-E64501, FLIR Systems Inc., USA) were employed to monitor the real-time temperature of varying concentrations of Ag@C (0, 20, 40, 80, and 160 µg/mL) under irradiation with 808 nm NIR light (Intensity: 0, 0.5, 1.0, 1.5, and 2.0 W/cm^2^) for 5 min.

### Cell culture

The human immortalized epidermal cell line HaCaT was acquired from Beyotime (Shanghai, China), while the mouse mononuclear macrophage cell line RAW264.7 was obtained from Procell (Wuhan, China). The cells were cultured in DMEM supplemented with 10% FBS and 1% P/S, and maintained at 37 °C in a humidified incubator with an atmosphere of 5% CO_2_.

### Cytotoxicity assessment

The cytotoxicity of Ag@C nanoparticles was evaluated by performing CCK-8 assays in HaCaT and RAW264.7 cells [23]. Cells were cultured in a 96-well plate with 100 µL of complete medium (CM) at a density of 2 × 10^4^ cells per well. After an overnight incubation period, the cells were treated with samples at varying concentrations of 0, 5, 10, 20, 40, and 80 µg/mL for 24 h. Following treatment, the medium was replaced with CM supplemented with a CCK-8 solution at a final concentration of 10%, and the cells were incubated for an additional time frame of between 1 and 4 hours at a temperature of 37 °C. Wells without any cells served as negative controls while those containing nanoparticles acted as positive controls. The optical density (OD) of the supernatant was subsequently measured at an absorbance wavelength of 450 nm using a Synergy HT microplate spectrophotometer (BioTek; VT, USA).

The cytotoxic effect of Ag@C on cells was further assessed using flow cytometry (FCM). Cells were initially cultured at a density of 1 × 10^5^ cells per well in a 12-well plate for 12 h. The experimental group received varying concentrations of Ag@C, while the control group remained untreated. Cells were then incubated for an additional 24 h. Following incubation, the cells were centrifuged at 1200 rpm for 5 minutes to remove the supernatant. Subsequently, the cells were stained and labeled according to the instructions provided with the Annexin V-FITC/PI apoptosis kit. Following this, detection was performed using a Sony SA3800 flow cytometer (FCM; Sony, Tokyo, Japan), and analysis was conducted with FlowJo software. For cell live/dead imaging, cells treated as described above were stained using the Calcein-AM/PI assay kit. Imaging was performed with a Nikon A1 confocal laser scanning microscope (CLSM; Nikon, Tokyo, Japan).

### Hemocompatibility assay

The murine blood samples were obtained from BALB/c mice (female, 6 ~ 8 weeks) which were purchased from Shanghai Laboratory Animal Research Center (Shanghai, China). Subsequently, 2 mL blood samples were mixed with 5 mL of PBS and centrifuged (2000 rpm, 5 min, 4 °C) to isolate the red blood cells (RBCs). The collected RBCs were washed 3 times with PBS and resuspended in 10 mL of PBS. A mixture of 100 µL of RBCs and 400 µL of Ag@C nanoparticles at varying concentrations was prepared [24]. Tubes containing 400 µL of PBS was utilized as the negative control, whereas water was employed as the positive control. After incubating the mixtures at 37 °C for a duration of 2 h, centrifugation was performed to isolate the supernatant. The absorbance of the supernatant was recorded at 570 nm utilizing the microplate reader.

### Bacterial culture

Methicillin-resistant Staphylococcus aureus (MRSA, ATCC 43300), which had been previously stored at -80 °C, was inoculated onto LB agar plates and incubated for 12 h at 37 °C in an incubator [25]. Single colonies of MRSA were subsequently selected from the plates and transferred to 5 mL of LB medium, thoroughly mixed, and allowed to incubate overnight at 37 °C on a shaker set to 220 rpm. The bacterial suspension was subjected to washing with normal saline, and the bacterial concentration was assessed by measuring the optical density at 600 nm. At an OD600 of 0.1, the corresponding bacterial concentration was approximately 10^8^ CFU/mL.

### Evaluation of anti-bacterial properties

Minimum inhibitory concentration (MIC) and minimum bactericidal concentration (MBC) were determined using the broth microdilution method [26]. Briefly, Ag@C nanoparticles were serially diluted in LB broth to final concentrations ranging from 1 to 160 µg/mL. MRSA suspensions were adjusted to 5 × 10^5^ CFU/mL and added to each well (100 µL bacterial suspension + 100 µL Ag@C dilution), with LB broth alone (negative control) and MRSA suspension without Ag@C (growth control) included. After incubation at 37 °C for 24 h, the MIC was defined as the lowest Ag@C concentration that completely inhibited bacterial growth. For MBC determination, 10 µL of bacterial suspension from wells with no visible growth was plated onto LB agar plates, incubated at 37 °C for 18 h, and the MBC was recorded as the lowest Ag@C concentration that reduced the bacterial count by ≥ 99.9% compared to the growth control.

For quantitative assessment of photothermal antibacterial activity, MRSA was washed with saline and diluted to a concentration of 2 × 10^6^ CFU/mL. Saline or Ag@C (80 µg/mL) was incubated with the MRSA dilution for 1 hour at 37 °C. The samples irradiated by NIR were exposed to an 808-nm laser at a power density of 1 W/cm² for 5 min [27]. Saline served as a negative control. The bacterial fluid mixtures from different treatments were further diluted in normal saline by factors of 10^1^, 10^2^, 10^3^, and 10^4^, after which 100 µL of each dilution was plated onto LB agar plates using a disposable coating rod. Following incubation at 37 °C for 18 h, colony-forming units (CFUs) were counted [28]. 

## Results and discussion

### The Preparation and characterization of Ag@C

**Fig. 2:**
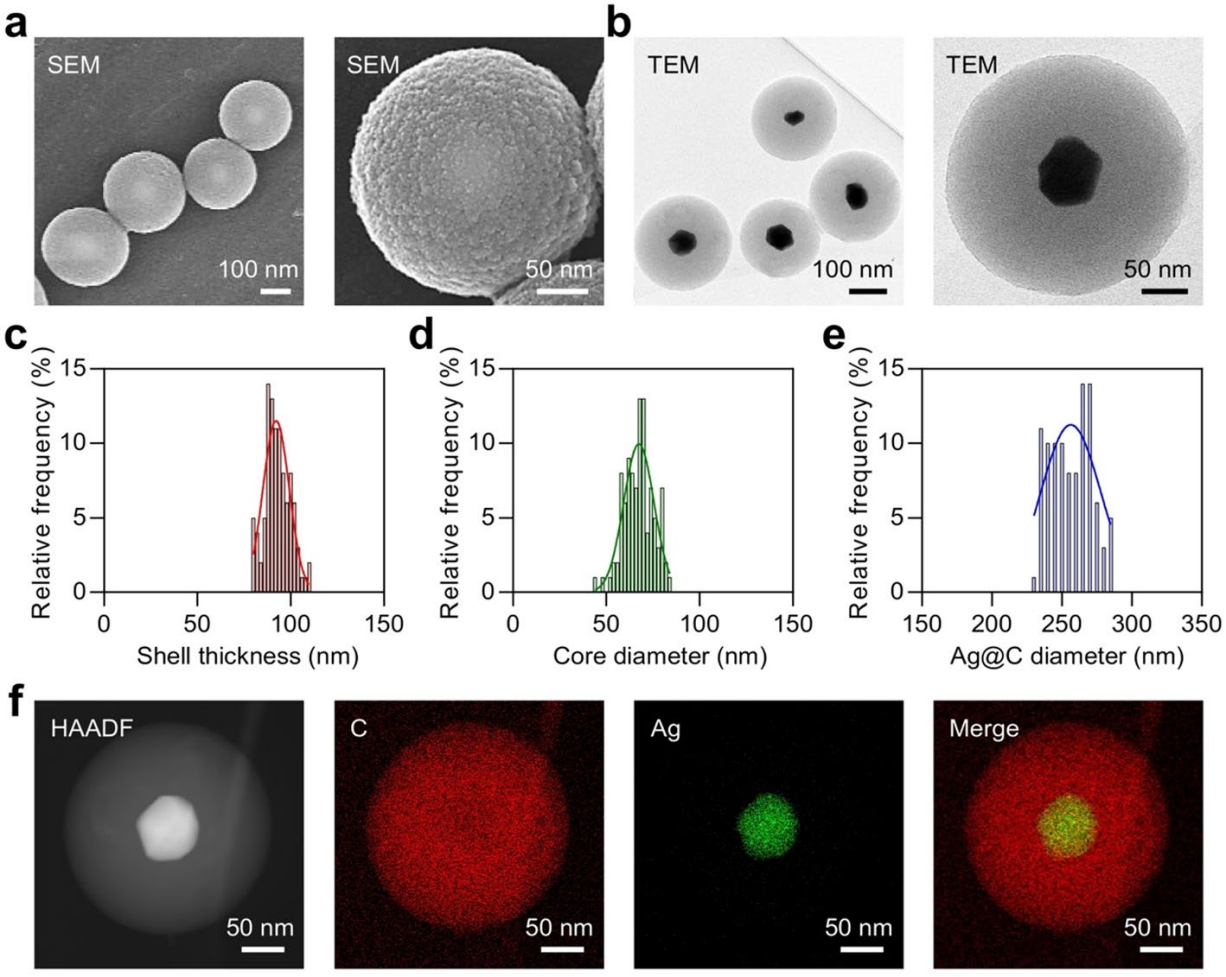
**a** SEM images of Ag@C. **b** TEM image of Ag@C. **c-e** C shell thickness distribution **c**, Ag core diameter distribution **d**, and total diameter distribution of Ag@C **(e)**. **f** High-angle annular dark-field (HAADF) and elemental mapping images of Ag@C. Scale bars, 50 nm

The formation of Ag@C core-shell nanospheres involves two primary steps (Fig. 2a): the nucleation of Ag nanoparticles in the core and the subsequent epitaxial growth and thickening of a carbonaceous shell around the Ag core. When the mixed solution of AgNO_3_ and glucose was subjected to heating in an autoclave, AgNO_3_ underwent reduction by glucose, leading to the nucleation of Ag nanoparticles. These nanoparticles are uniformly dispersed within the solution, and their outer surface exhibits catalytic activity for the carbonization of glucose. This process results in the in-situ deposition of carbonaceous products on the surface of the Ag nanoparticles, forming a carbonaceous shell [22]. 

**Fig. 3:**
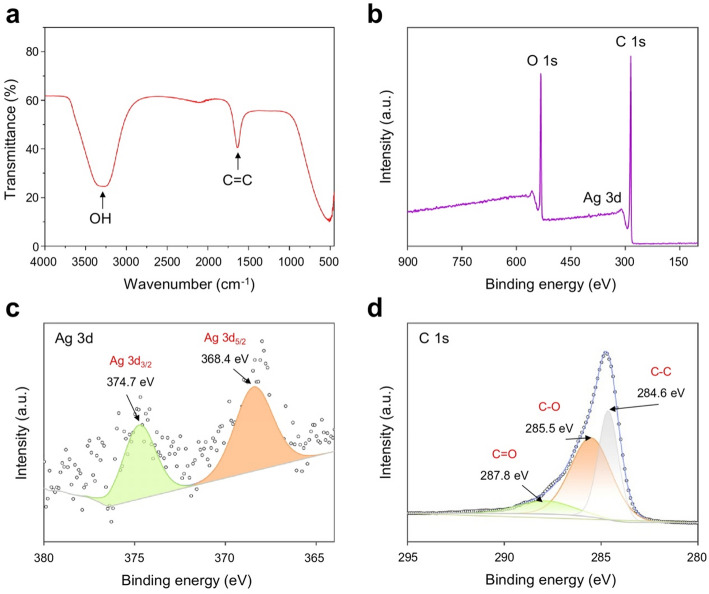
**a** FTIR spectra of the Ag@C. **b-d** XPS analysis of Ag@C **b**, detailing Ag 3d **(c)** and C 1s **(d) **regions

SEM and TEM images revealed that Ag@C exhibited a distinct core-shell structure characterized by globular morphology (Fig. 2a and b). Based on the TEM images, the measurements for shell thickness, core diameter, and overall diameter of Ag@C were determined to be 92.20 ± 7.03, 67.45 ± 8.13, and 256.40 ± 21.13 nm, respectively (Figs. 2c-e). The energy dispersive X-ray (EDX) elemental mapping further indicated that Ag was predominantly located in the core of Ag@C, while C was primarily found in its outer shell (Figs. 2f and S1). Fourier transform infrared spectroscopy (FTIR) revealed the presence of numerous functional groups, such as −OH and = C = C, within the carbon framework of the carbonaceous component (Fig. 3a). X-ray photoelectron spectroscopy (XPS) showed characteristic peaks corresponding to Ag 3d, C 1s, and O 1s (Figs. 3b-d and S2). The binding energies for Ag 3d were measured at 368.4 eV and 374.7 eV, which correspond to the Ag 3d5/2 and Ag 3d3/2 states, respectively (Fig. 3c).

**Fig. 4:**
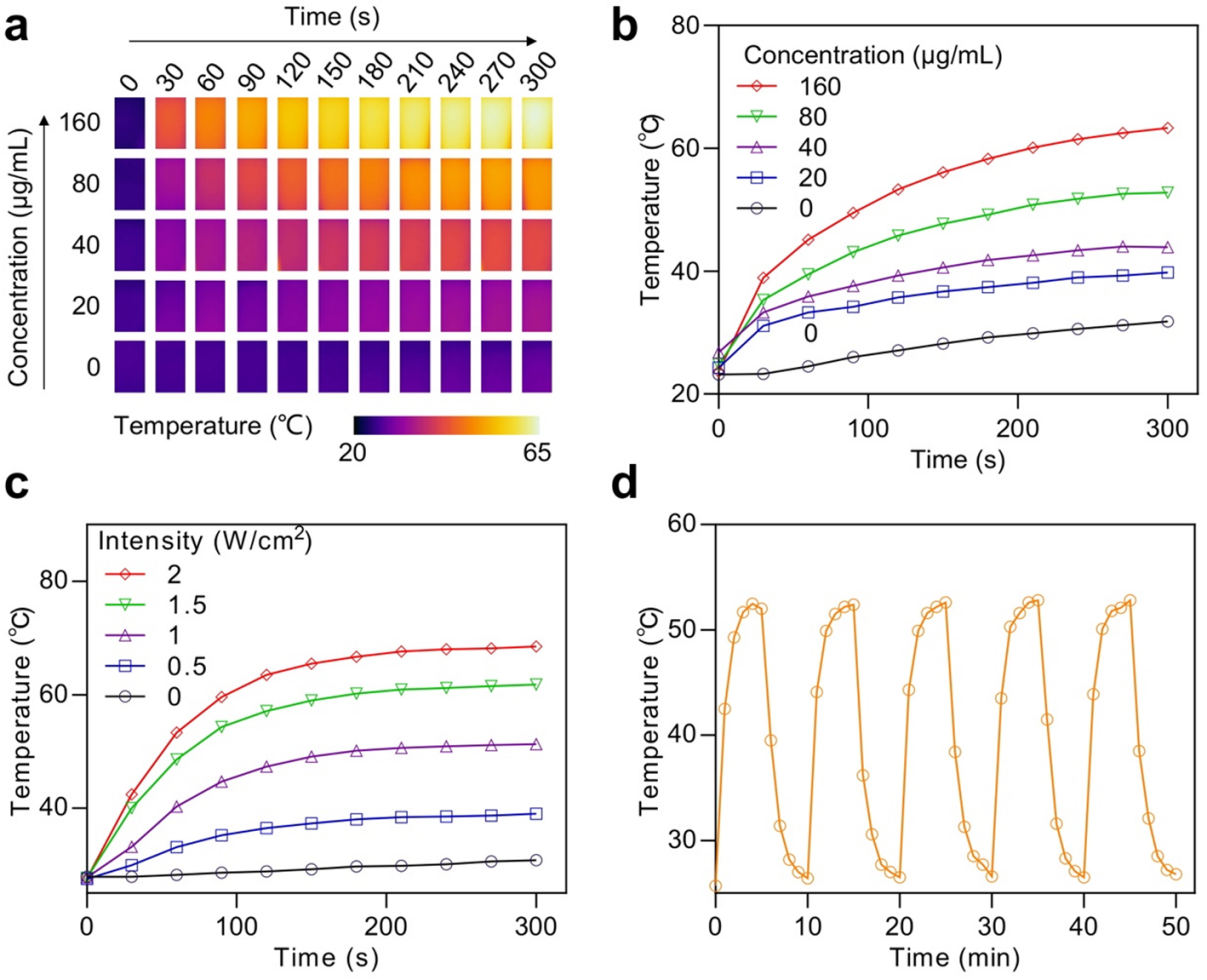
**a** Thermal infrared images and **b** photothermal response curves of Ag@C suspensions at concentrations of 20, 40, 80, and 160 µg/mL under 808 nm NIR laser irradiation at 1.0 W/cm^2^. **c** Temperature profiles of Ag@C (80 µg/mL) under varying laser power densities. **d** Photothermal stability curves of the Ag@C solution demonstrated through laser on/off cycles at 1.0 W/cm^2^

The absorbance spectrum shows that Ag@C nanoparticles exhibit a broad and strong absorption band in the NIR region (700–1000 nm), with an absorption peak centered at ~ 808 nm (Figure S3), suggesting the correlation between NIR absorption and photothermal activity. Therefore, we subsequently employed a thermal imager to assess the photothermal conversion efficiency of Ag@C under near-infrared (NIR) irradiation. Initially, we recorded the temperature increase and corresponding heat map for various concentrations of Ag@C subjected to 808 nm laser irradiation at 1 W/cm² (Fig. 4a and b). The results indicated that the temperature of an 80 µg/mL Ag@C solution rose by 28.0 °C over 5 min *in vitro*, while the control group with H_2_O exhibited only an increase of 8.6 °C. Furthermore, the temperature of the Ag@C solution was influenced by laser power; at a lower power setting (0.5 W/cm²) for 5 min, the temperature increased by just 11.5 °C, whereas at higher power (2.0 W/cm²), it surged by 40.9 °C (Fig. 4c). Even after 5 alternating cycles of switching lasers, Ag@C demonstrated remarkable photothermal stability, with no significant change in its maximum transition temperature (Fig. 4d). These findings suggest that our synthesized Ag@C exhibits excellent photothermal performance.

### The *in vitro* biocompatibility of Ag@C

**Fig. 5:**
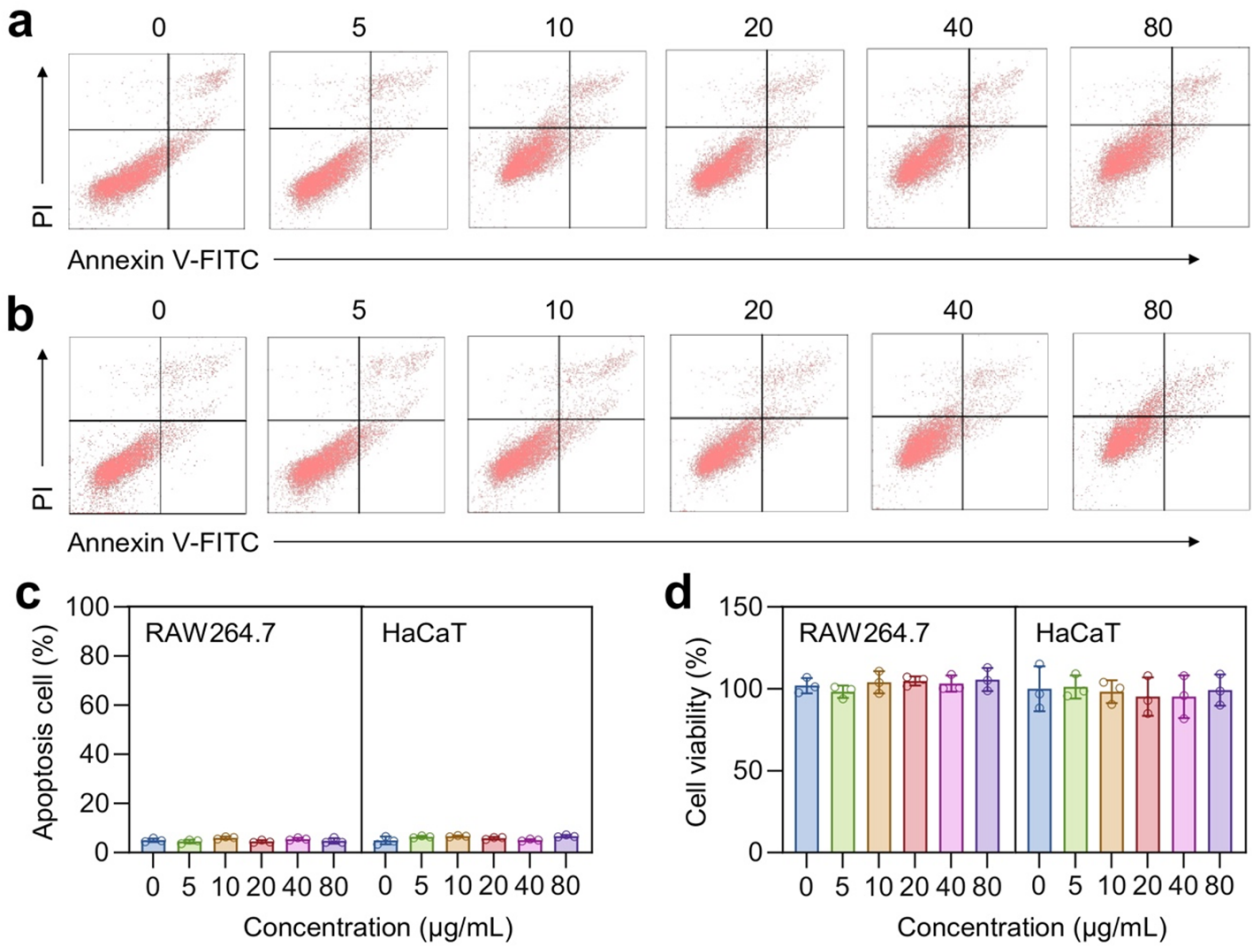
**a**,** b** Representative FCM plots of apoptotic RAW264.7 **(a)** and HaCaT **(b)** cells after incubating with Ag@C at concentration ranging from 5 to 80 µg/mL for a duration of 24 h. **c** Quantitative FCM analyses of apoptotic RAW264.7 and HaCaT cells with different treatments. **d** Evaluation of biocompatibility using CCK-8 assay

**Fig. 6:**
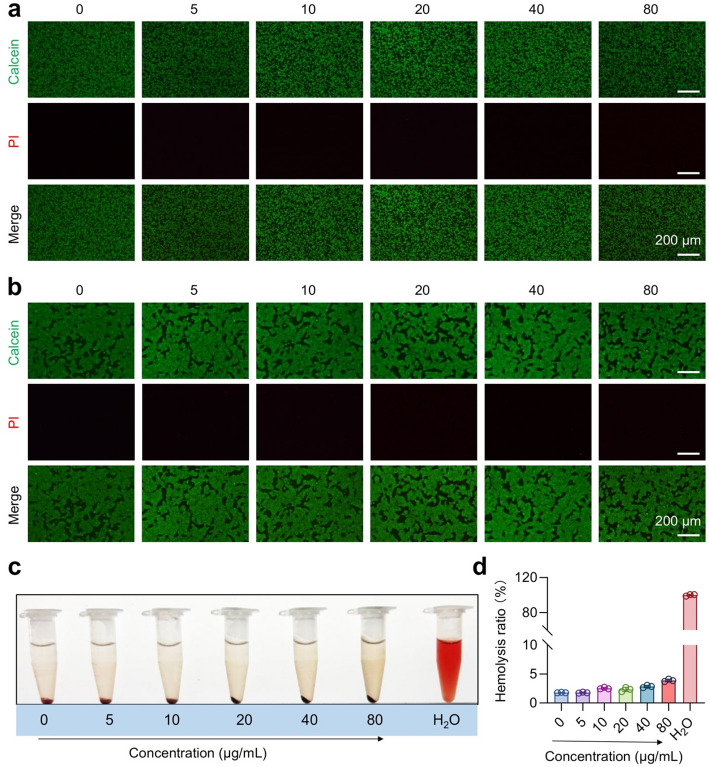
**a**,** b** CLSM images of Calcein-AM and PI co-stained RAW264.7 **(a)** and HaCaT **(b)** cells after incubation with Ag@C at varying concentrations. Scale bars, 200 μm. **c**,** d** Representative images **(c)** and hemolysis ratio **(d)** of freshly isolated erythrocytes incubated with Ag@C at concentration ranging from 5 to 80 µg/mL for a duration of 2 h

To evaluate the biocompatibility of the synthesized Ag@C, we initially assessed the apoptosis of RAW264.7 and HaCaT cells treated with various concentrations (5, 10, 20, 40, and 80 µg/mL) of Ag@C using flow cytometry (FCM). The results from FCM indicated that Ag@C exhibited excellent biocompatibility with both RAW264.7 and HaCaT cells (Fig. 5a and b). Even at a concentration of 80 µg/mL, the number of apoptotic cells did not show a statistically significant difference compared to the control group (Fig. 5c). The *in vitro* cytotoxicity of Ag@C against RAW264.7 and HaCaT cells was then assessed using the CCK-8 assay. Even at a concentration of 80 µg/mL for 24 h, Ag@C exhibited minimal cytotoxicity (cell viability > 90%) towards both cell types (Fig. 5d), which aligns with the findings from the apoptosis assay conducted via FCM. The biocompatibility of Ag@C was further validated through calcein AM/propidium iodide (PI) staining. CLSM images demonstrated that the viability of RAW264.7 and HaCaT cells co-cultured with varying concentrations of Ag@C remained largely unaffected, with no significant cell death observed (Fig. 6a and b). Hemolysis experiments were conducted using mouse red blood cells, revealing that Ag@C induced minimal to no hemolysis even at nanoparticle concentrations as high as 80 µg/mL (Fig. 6c and d), which indicates its excellent blood compatibility. Collectively, these findings suggest that Ag@C exhibits excellent biocompatibility and holds promise as an antibacterial vector for potential clinical applications.

To assess the long-term biocompatibility of Ag@C, we performed the CCK-8 assay to evaluate the viability of RAW264.7 cells over extended time periods (24, 36, and 48 h) upon treatment with Ag@C nanoparticles, alongside bare AgNPs with equivalent Ag content as a control. As shown in Figure S4, the long-term cell viability data revealed that Ag@C maintained excellent biocompatibility even over 72 h. The key reason for the significantly reduced cytotoxicity of Ag@C relative to bare AgNPs lies in the physical isolation and regulation effect of the carbonaceous shell. Bare AgNPs tend to continuously release Ag⁺ in biological environments, and the excessive accumulation of free Ag⁺ is a major cause of cytotoxicity [29]. The carbonaceous shell of Ag@C acts as a robust physical barrier, which tightly wraps the Ag core and restricts the uncontrolled and sustained release of Ag⁺. This “controlled release” pattern avoids the toxic effects caused by its overaccumulation in mammalian cells while maintaining the antibacterial activity of Ag⁺.

While Ag-based nanoparticles exhibit advantages such as a broad spectrum of activity, high efficiency, and low resistance to antimicrobial agents, their potential cytotoxicity poses limitations for *in vivo* applications. It has been reported that the continuous release and long-term accumulation of Ag^+^ ions in bodily fluids are primary contributors to the cytotoxic effects associated with silver nanoparticles [30]. Our findings suggest that the biocompatibility of silver nanoparticles can be significantly enhanced by incorporating a carbonaceous shell around them, likely due to the physical isolation and protective properties provided by this shell against the silver core.

### Antimicrobial capacity of Ag@C

**Fig. 7:**
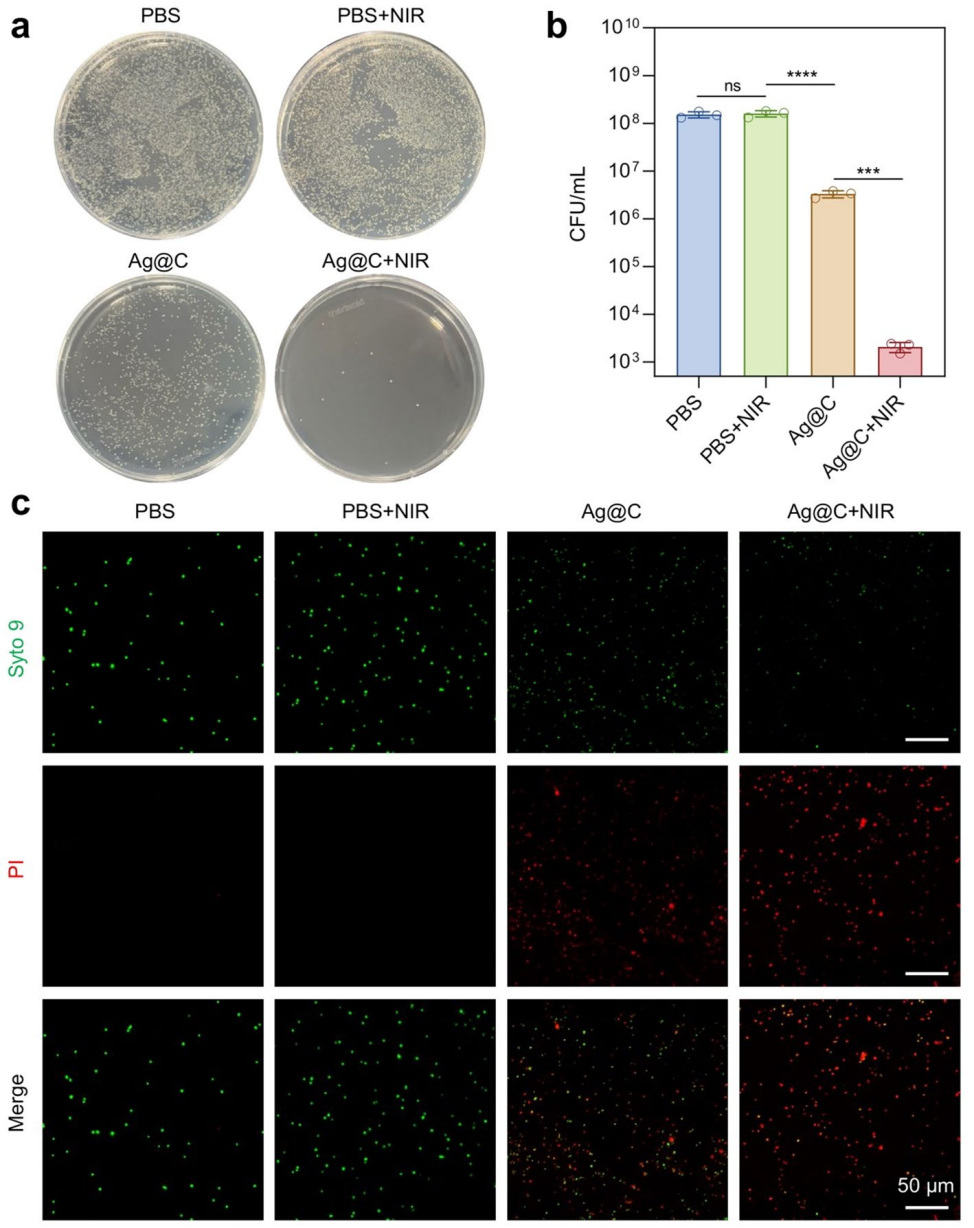
**a** Photographs of bacterial broth plates of MRSA post-treatment with PBS, PBS + NIR, Ag@C, and Ag@C + NIR. **b** Colony-forming units (CFUs) count of MRSA with different treatments. **c** CLSM images of MRSA with different treatments. Scale bars, 50 μm

As bacterial resistance to antibiotics continues to rise, the effectiveness of traditional antibiotics is gradually diminishing. Silver-based nanoparticles have garnered significant attention due to their broad-spectrum antibacterial properties and low levels of resistance. We subsequently employed MRSA to assess both the inherent antibacterial capability of Ag@C and its photothermal antibacterial efficacy in response to NIR light. As illustrated in Fig. 7a, Ag@C (80 µg/mL) effectively inhibited the growth of MRSA on its own, furthermore, bacterial cultures treated with Ag@C and exposed to NIR irradiation exhibited a significant reduction in colony counts on AGAR plates (Fig. 7b). The bacteria subjected to the combined treatment of Ag@C and near-infrared light showed markedly lower survival rates compared to those treated with either NIR alone or Ag@C alone, indicating a synergistic antibacterial effect. Additionally, we employed the green SYTO 9 fluorescent dye, which penetrates both intact and damaged bacteria, alongside the red PI dye that selectively enters only damaged bacterial membranes. This approach was utilized for live/dead bacterial viability assays, with CLSM imaging applied to elucidate the antibacterial effect of Ag@C under NIR irradiation. CLSM images indicated that the red fluorescence of MRSA treated with PBS and PBS + NIR was nearly undetectable, while a moderate level of red fluorescence was observed in the Ag@C treatment group (Fig. 7c). The highest proportion of dead bacteria exhibiting red fluorescence was found in the Ag@C + NIR group, further demonstrating that bacteria treated with Ag@C displayed enhanced bacterial killing efficiency due to the strong synergistic effect between Ag@C and photothermal treatment.

The carbonaceous shell not only enhances safety by isolating silver ion release but also imparts photothermal capabilities to Ag@C, enabling it to respond effectively to NIR irradiation [31]. PTT represents a novel therapeutic strategy that harnesses photothermal agents to convert light energy into heat in response to external illumination [32]. PTT is characterized by its high selectivity, low invasiveness, and absence of toxic side effects. Currently, it has emerged as a promising antibacterial strategy [33]. Despite the significant potential of PTT in addressing bacterial infections, the efficacy of PTT alone for antibacterial treatment remains suboptimal due to limitations associated with most photothermal agents, such as poor photostability, low photo-absorption rates, and inadequate photothermal conversion efficiency [7]. Our findings indicate that Ag@C exhibits excellent photothermal properties and stability, along with favorable photothermal conversion efficiency, making it a superior candidate for use as a photothermal therapeutic agent.

## Conclusion

In this study, we successfully developed Ag@C core-shell nanoparticles as a novel antibacterial agent with enhanced biosafety and photothermal therapeutic efficacy. The Ag@C nanoparticles exhibited a well-defined core-shell structure, favorable optical properties for NIR-triggered PTT, and negligible cytotoxicity both *in vitro* and in hemocompatibility assays. The synergistic combination of Ag@C and NIR irradiation demonstrated significant eradication of MRSA, achieving superior antibacterial effects. Notably, the carbon shell effectively mitigated the inherent cytotoxicity of silver cores, addressing a critical limitation of traditional silver-based nanoparticles. These findings highlight the potential of Ag@C + NIR as a dual-functional platform for precise antibacterial intervention, combining targeted photothermal ablation with minimized off-target effects. Future studies should focus on *in vivo* validation, long-term degradation tracking, long-term biosafety evaluation, and scalability of synthesis to advance this technology toward clinical applications. Overall, Ag@C-based PTT represents a promising strategy for combating multidrug-resistant bacterial infections while prioritizing patient safety.

## Supplementary Information

Below is the link to the electronic supplementary material.

Supplementary Material 1

## Data Availability

All data that support the findings of this study are included within the article (and any supplementary files).
