# Supplementary material for: Synergistic photothermal antibacterial efficacy and high biocompatibility of silver-carbon core-shell nanoparticles
Source: Discov Nano. 2026 Jan 16;21(1):9. doi: 10.1186/s11671-026-04432-w (PMC12811206; doi:10.1186/s11671-026-04432-w)
Supplement: Supplementary file 1 — Supplementary Material 1 [file 11671_2026_4432_MOESM1_ESM.docx]

**Supporting Information**

**Synergistic Photothermal Antibacterial Efficacy and High Biocompatibility of Silver-Carbon Core-Shell Nanoparticles**

Chunning Gu,^1^ Li Guo,^1^ Ziqian Zhou,^1^ Anyuan Shi,^1^ Lele Wu,^1,^* Wei Cheng^1,^*

^1^Nanjing Stomatological Hospital, Affiliated Hospital of Medical School, Institute of Stomatology, Nanjing University, 30 Zhongyang Road, Nanjing, Jiangsu 210008, China.

Email: 18816214023@163.com (Lele Wu) and dentist_nj@163.com (Wei Cheng)


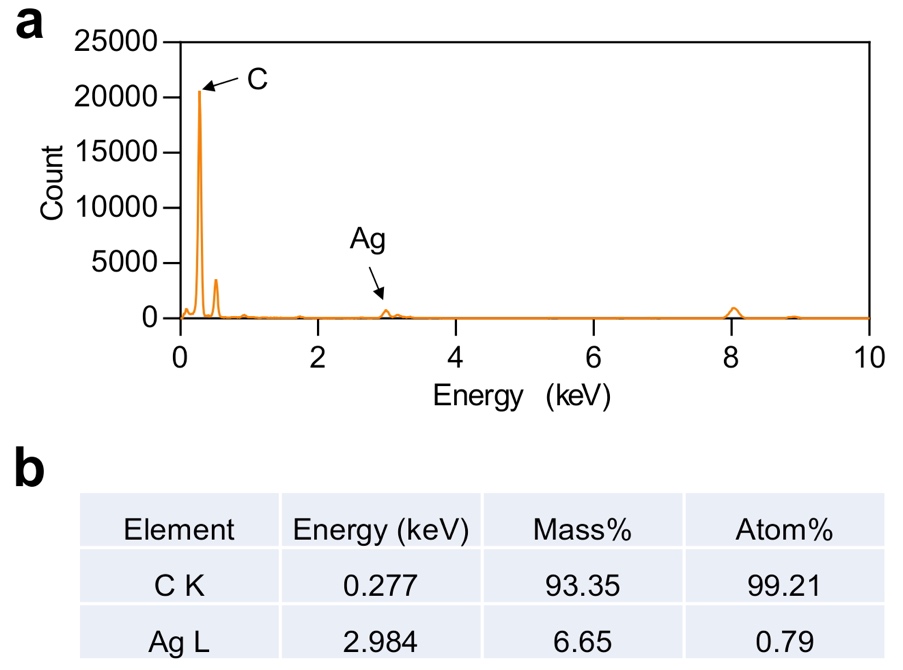


**Figure S1. a)** Energy dispersive X-ray (EDX) spectrum of the Ag@C. b) Proportions of C and Ag elements in Ag@C nanoparticles.


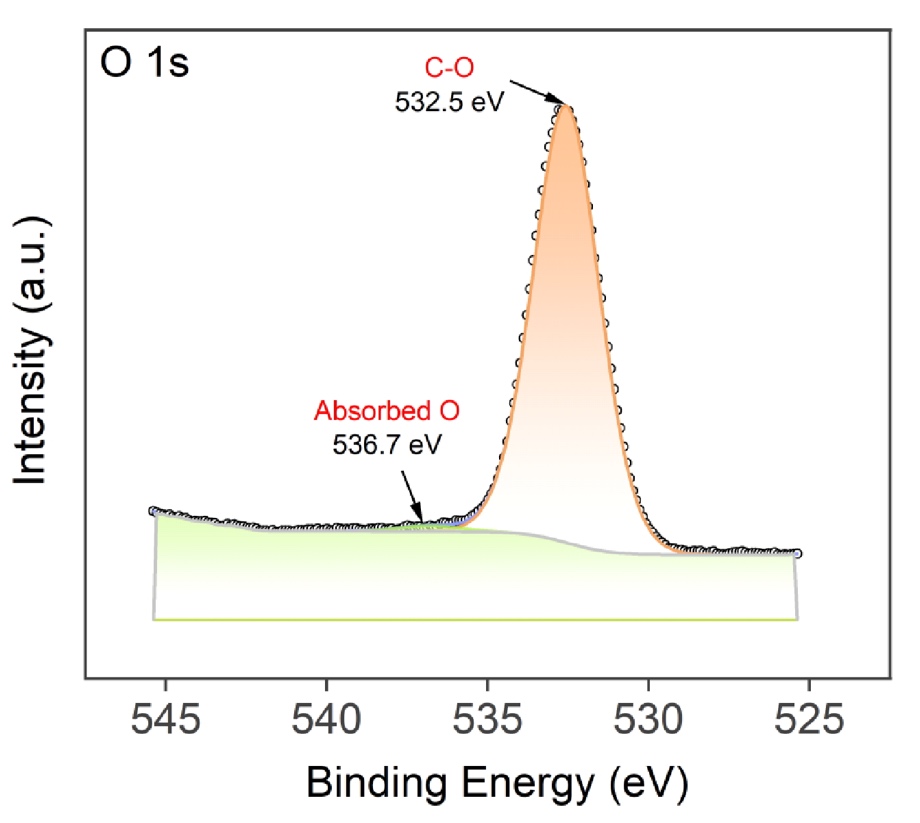


**Figure S2.** X-ray photoelectron spectroscopy (XPS) analysis of Ag@C detailing O 1s region.


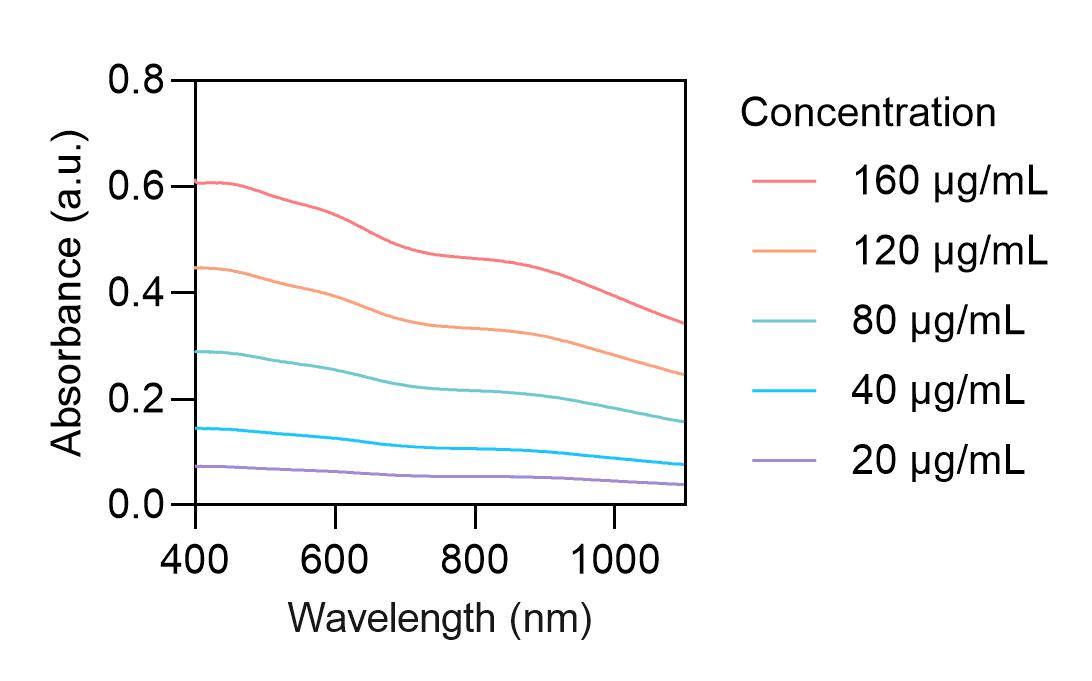


**Figure S3.** Ultraviolet-visible-near-infrared (UV-Vis-NIR) absorbance spectrum of Ag@C nanoparticles.


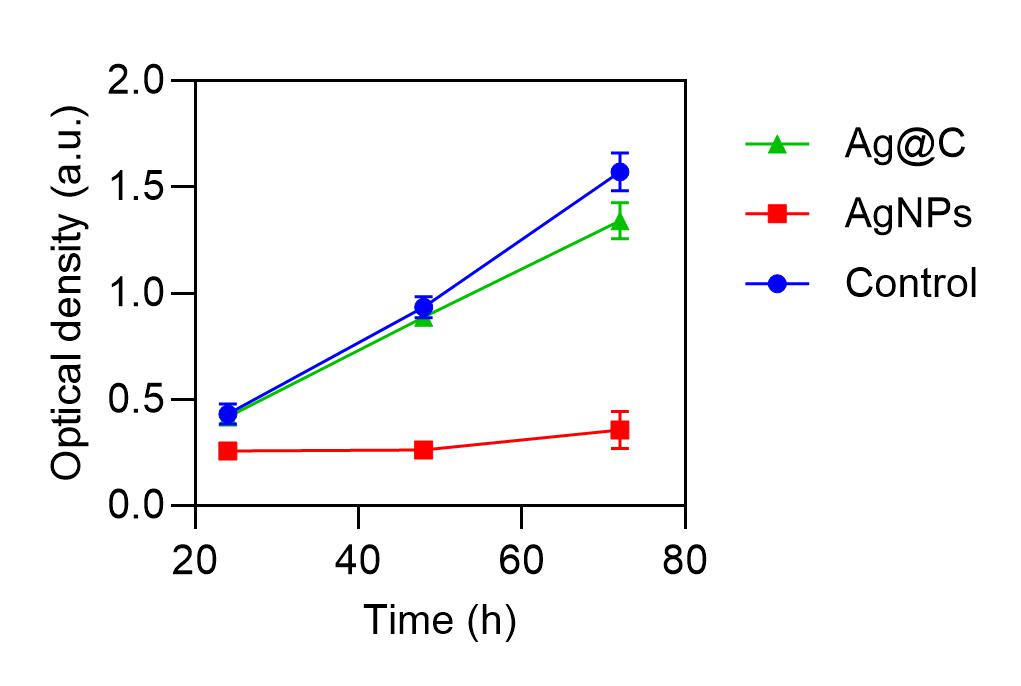


**Figure S4.** The optical density values of RAW264.7 cells co-cultured with PBS, Ag@C (80 μg/mL), and AgNPs (equivalent to Ag@C) for 24, 48, and 72 hours, as assessed by CCK8 assay.
